# Network Structure and City Size

**DOI:** 10.1371/journal.pone.0029721

**Published:** 2012-01-12

**Authors:** David Levinson

**Affiliations:** Department of Civil Engineering, University of Minnesota, Minneapolis, Minnesota, United States of America; University of Zaragoza, Spain

## Abstract

Network structure varies across cities. This variation may yield important knowledge about how the internal structure of the city affects its performance. This paper systematically compares a set of surface transportation network structure variables (connectivity, hierarchy, circuity, treeness, entropy, accessibility) across the 50 largest metropolitan areas in the United States. A set of scaling parameters are discovered to show how network size and structure vary with city size. These results suggest that larger cities are physically more inter-connected. Hypotheses are presented as to why this might obtain. This paper then consistently measures and ranks access to jobs across 50 US metropolitan areas. It uses that accessibility measure, along with network structure variables and city size to help explain journey-to-work time and auto mode share in those cities. A 1 percent increase in accessibility reduces average metropolitan commute times by about 90 seconds each way. A 1 percent increase in network connectivity reduces commute time by 0.1 percent. A 1 percent increase in accessibility results in a 0.0575 percent drop in auto mode share, while a 1 percent increase in treeness reduces auto mode share by 0.061 percent. Use of accessibility and network structure measures is important for planning and evaluating the performance of network investments and land use changes.

## Introduction

The average American spends about 4 years of their life in motion. The amount depends on who they are, what they do, where they live, and how they choose to travel. Most Americans live in metropolitan areas that enable people to engage in the activities they care about efficiently, by bringing activities and people close together for mutual economic production, trade, and commerce, social interaction, education, and defense. This proximity (accessibility) must provide advantages, otherwise cities would not exist. But not all cities are equally efficient. They vary in size and scope, they vary in the density and location of activities, and they vary in their internal circulatory systems that enable people to move between places. As the world continues to urbanize, even small gains in intra-urban organizational efficiency will lead to large gains for humanity as a whole.

The structure of urban networks shapes the efficiency of the cities they serve. While in general there are many characteristics that scale with city size (metropolitan population (the terms “cities” and “metropolitan areas” are used synonymously in this paper)), not all cities are created equal. They grew under different technological, political, and legal regimes and operate in different physical environments, and as a consequence manifest different physical forms.

A recent book *The Triumph of the Cities* [1] has publicized what had been heretofore an academic debate about the efficiency of cities, both in reduced infrastructure costs per capita, and in increased productivity. There is a modest literature examining the inputs to cities, how do network structure and urban services vary across cities. This has been examined for metro systems, [2]–[5], roads [6]–[11], and other facilities [12]–[14].

There is also a large and growing literature examining the outputs from cities: how productive are cities, do they generate agglomeration economies, GDP, patents, and if so, how large is their agglomeration benefit. The literature finds that larger cities produce more GDP per capita, more patents, and more innovation, though there are of course debates about magnitudes. [15]–[30].

The travel behavior literature shows that larger cities have more congestion and longer commutes, which implies inefficiency, even if those commutes are not increasing as fast as population growth [31]. However if those longer commutes result in better jobs (a better match of worker skills to employer needs), and that congestion is the result of non-work travel caused by expanded consumption (goods that better fit desires) [32], then those implied inefficiencies of transportation are simply the product of choices that urban consumers make that is dominated by the benefits that created them [33]. After all, people could choose to have shorter commutes [34], or to consume fewer specialist goods and services, even if they lived in a large city.

This paper compares networks across cities, examining relationships between the macro (overall system performance) and averages of micro measures (network structure) with the aim of discovering key relationships that might be used to inform future network designs. It focuses on the questions of how network scale and connectivity vary with city size. This connectivity that cities enable, and of which networks determine efficiency, may drive the expanded outputs of larger cities noted above. On the one hand, larger cities consume more area, which makes connectivity more difficult, on the other, they increase population density, requiring more connected networks to serve. Whether connectivity increases is in the end an empirical question.

The authors have previously examined how network structure affects transportation performance (congestion, travel per person) [35]. This paper considers how accessibility, network structure, and city size affects other measures of transportation performance: journey-to-work time and automobile mode share. It has been hypothesized that network connectivity increases with city size as the value of the increased access outweighs the costs of building the additional links [36].

This research posits that network connectivity increases with metropolitan population. Network connectivity is created by agents (land developers, governments) who build network links to connect places to the network [36]. All places must have at least one connection to the network (i.e. there must be at minimum a tree connecting developed land parcels). However, there may be some value to network builders to create cross connections (circuits) so that the network becomes more web-like. The advantage of the additional links is reducing travel costs compared to trees, the disadvantage is the additional construction costs. That value is determined by the accessibility the additional connection creates.

In short, this model predicts that road networks will be more connected, less circuitous, and less tree-like the greater the accessibility a new link creates. Accessibility by road increases with population (i.e. more people can be reached in a given time the larger (denser) the city is) if density increases accessibility more than the resulting congestion and decline in average network speed decreases it. This will be true if there is excess road capacity, or if there are non-road modes of transportation (e.g. metro systems) which serve travelers when roads are congested [37]–[39] thereby limiting the amount of road congestion, and perhaps in other conditions. Thus larger cities have a greater incentive for agents to build cross-connecting links since those links will be more valuable. These cross-connecting links in addition to reducing travel distances compared with dendritic networks also may relieve congestion on the network. If private developers are building links, their requirement is that the embedded land value of the accessibility created by the new link exceeds the cost of link construction. Public agencies require that the public welfare created exceeds the cost of link construction. Previous research suggests publicly built networks have different development objectives than privately built ones [40].

This paper begins with a discussion of network characteristics. This is followed by an explanation of the data used. Summary statistics of how network structure varies with city size is presented. Next are scaling rules, which used in a systematic set of regression models to ascertain whether city scaling is linear, sublinear, or superlinear with population for a set of variables. This study calculates and compares accessibility across 50 US metropolitan areas. It then uses accessibility, network structure, and city size to explain journey-to-work travel time and automobile mode share. The discussion identifies some implications for urban planning.

## Methods

### Characterizing Networks

There are a variety ways of characterizing networks, developed in the field of transportation geography and network science, reviewed in [41]. Selected measures used in this paper are discussed below.

#### Connectivity

Transportation geography provides a classic set of connectivity measures [42].

The alpha index (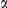) is the ratio of the actual number of circuits in a network to the maximum possible number of circuits on that planar network network. It is given as:
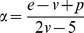
(1)where 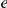 = number of edges (links), 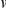 = number of vertices (nodes), 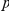 = number of graphs or subgraphs, and for a network where every place is connected to the network 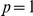.

Values of 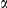 range from 0 percent – no circuits – to 100 percent – a completely interconnected network.

The beta index (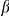) measures the connectivity relating the number of edges to the number of nodes. It is given as:
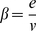
(2)


The greater the value of 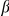, the greater the connectivity. As transport networks develop and become more efficient, the value of 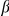 should rise.

A 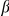 of 1.0 is a minimally connected network where the links form a cycle. If we limit junctions to 4 incoming links, (as is typical of urban intersections, with a few outliers) and all junctions were 4-way, we would get a 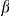 of 2.0 (each node has four two-way in-links).

The gamma index (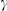) measures the connectivity in a network. It is a measure of the ratio of the number of edges in a network to the maximum number possible in a planar network
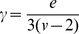
(3)


The index ranges from 0 (no connections between nodes) to 1.0 (the maximum number of connections, with direct links between all the nodes).

The eta index (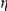) measure the length of the graph over the number of edges.
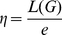
(4)


The theta index (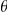) measure the traffic (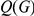) (e.g. system vehicle kilometers traveled) per vertex.
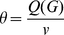
(5)


Most road networks have 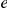 and 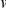 of similar orders of magnitude, so 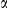, 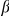, and 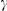, while differentiated for small networks, are highly correlated (correlation coefficient of approximately 1.0) for large networks.

#### Treeness

The treeness (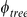) measure [43] is based on the two basic structures of a planar transportation network: circuit and tree [44]. A circuit is defined a a closed path, with no less than three links, that begins and ends at the same node. A tree is defined as a set of connected lines that do not form a complete circuit. Each link belongs to a branch or a circuit network. Open source software developed in [43] classified each link. This code was implemented on the street network of each metropolitan area.

The treeness for each street network is given as:
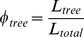
(6)where,


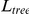
,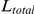 = Length of street segments belonging to a branch, entire network (km).

#### Circuity

Network circuity is defined as the ratio of the shortest path network distance to the Euclidean or straight line distance between an origin and destination, and captures the spatial (in)efficiency of the network in connecting two points. [45] used a dataset of randomly selected, origins and destinations of actual trips to estimate circuity in their analysis of commute patterns and compared that to random OD points, finding that circuity of actual home to work trips was lower than random OD points of the same trip length. The correlation coefficient between the circuity of actual home to work trips with random points constrained to match that trip length is 0.36 (Author's calculations). The mean circuity for actual home to work trips in 20 US metros with complete data is 1.18, while the mean point-to-point circuity for those same metros, constraining the average trip distance to the be same, is 1.26 (Author's calculations). Here we use circuity of random trips constrained to match actual trip length. This is driven by data availability. The US Census's Longitudinal Employment Household Dynamics dataset, which has actual origins and destinations, is only available for some metropolitan areas, and only since 2003.

For each metropolitan area in our dataset, two samples were generated. The first sample of 200 randomly distributed origins and the second sample of 1000 randomly distributed destinations were generated using GIS. This provided 200*1000 OD pairs for each area resulting in a 200,000 OD matrix. The network distance and the euclidean distance were calculated for each of 200,000 OD pairs.

A subsample of OD pairs were extracted from the 200,000 random OD matrix in each metropolitan area by matching the network distance to the average commute trip length, provided in the 2001 National Household Travel Survey (NHTS). The average circuity for the subsample of OD pairs in each area was then estimated as:
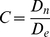
(7)where,


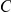
 = Average circuity


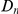
 = Sum of the network distance between all OD pairs in the subsample,


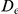
 = Sum of the euclidean distance between all OD pairs in the subsample.

#### Accessibility

Accessibility is defined as the ease of reaching valued destinations. For instance, how many jobs one can reach in 30 minutes by car in the morning peak. Accessibility varies by location, by time threshold (10 minutes, 30 minutes, 60 minutes), by time (hour of day, day of week, month of year, year), by mode of travel, and by type of destination (jobs, houses, shops, parks, schools). Accessibility indicates how well the transportation system serves its ultimate goal, moving people and freight to the destinations they care about.

**Figure 1:**
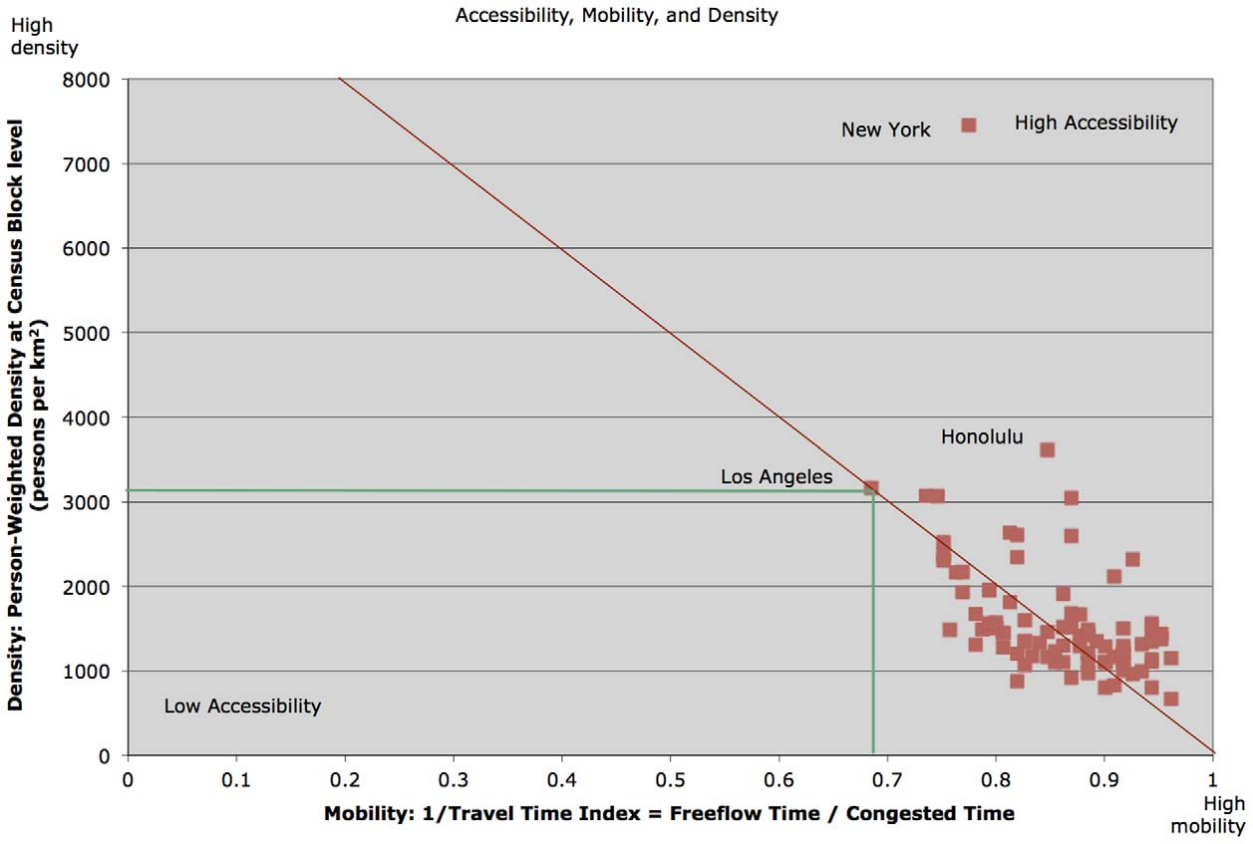
Mobility vs. Density.

Accessibility combines travel time on the network (which depends on speed and spatial structure: how well organized the network is) and activities (how many activities there are and how well they are located). Clearly there is a trade-off between these two factors. Cities with higher densities of activity (more people per unit area) tend to be slower. But cities vary in their internal organization, so some cities may construct more efficient internal plans and policies than others. An illustration of this trade-off is shown in Figure 1. The upper right corner of the graph is the region of high accessibility, the ability to move fast and reach lots of destinations, while the lower left corner is low accessibility, slow movement with no opportunities available. Most US cities cluster along a line, and as density increases, mobility decreases. New York is a notable exception, achieving a high accessibility because its density increases more than its mobility decreases, enabled by its powerful transit network.

The cumulative opportunity measure of accessibility 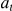 estimates the number of destinations that can be reached in a given time threshold (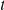) [46]. A measure for a metropolitan average accessibility below:
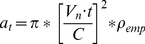
(8)where,


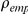
 = Urban area employment density (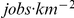).


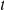
 = time threshold.


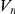
 = Average network velocity in 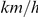



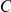
 = Average circuity, as estimated above

Accessibility 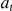 (in the regressions 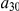 is used) was estimated for each study area using a combination of the above estimated circuity and the employment density of the urbanized area in (persons/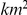), along with network speed, but is constrained not to exceed metropolitan area employment 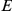:
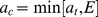
(9)


The weighted average of accessibility discounts long time thresholds more than short thresholds. Here we difference time thresholds to get a series of donuts (e.g. Jobs reachable from 0 to 10 minutes, from 10 to 20 minutes, etc.).
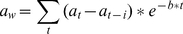
(10)where 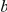 = −0.08 based on previous work [47], and 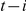 denotes the next smaller time threshold.

#### Entropy

Road networks are heterogeneous, considering the differentiated functional designs and operational performance of hierarchical roads.

The entropy measure of heterogeneity is given as:
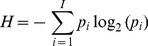
(11)Where 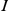 is the number of subsets in the system, and 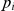 is the proportion of elements in the 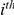 subset.

Individual links can be grouped into subsets based on different road properties such as functional type, traffic volume, capacity, or level of service. In this paper, entropy is defined by functional class.

### Data

#### Street Networks

The street networks for the fifty metropolitan areas, used in this analysis, were extracted from the Census TIGER/line files. The extracted networks for the metropolitan areas were cleaned to include just the road features based on the Feature Class Codes (FCC) for the line segments provided in the Census TIGER/Line files. They were further cleaned using TransCAD software to eliminate nodes which served no topological purpose, and to combine the resulting links.

#### Travel Data

Travel data from the Texas Transportation Institute's Urban Mobility Report [48] provides information on the long-term congestion trends and the most recent congestion comparisons for 90 urban areas across the U.S. Journey to work times are derived from the American Community Survey (2005–2008).

#### Socio-Demographic Data

The socio-demographic data was obtained for the year 2010 from the U.S Census Bureau for the fifty metropolitan areas considered in the analysis (listed in Table 3).

## Results

### Network Variations with City Size

Most network structures vary systematically over a large range of metropolitan areas (from about 1 million to 18 million persons) under the current technology state of automobiles on roads. Table 1 summarizes a variety of network structure statistics by metropolitan population quintile (quintile 5 represents the 10 largest US metros, quintile 4 the next 10 largest, and so on) for the 50 largest metropolitan areas in the US. The use of quintiles is illustrative and not intended to suggest any particular process for which quintiles are an organizational feature of the system of cities.

**Table 1:** Mean Network Structure Variables by Metropolitan Population Quintile.

	Smaller				Larger
Variable	1	2	3	4	5
alpha	0.14	0.14	0.16	0.18	0.17
beta	1.27	1.29	1.31	1.36	1.35
gamma	0.42	0.43	0.44	0.45	0.45
eta ( 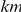 )	0.07	0.07	0.09	0.09	0.11
theta	0.53	0.62	0.76	0.91	1.12
Circuity	1.32	1.31	1.33	1.33	1.27
Treeness	0.29	0.26	0.26	0.23	0.21
Entropy	0.60	0.54	0.42	0.34	0.42
Roadways per capita ( 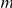 )	7.1	6.6	6.4	5.5	5.4
Delay (%)	14	22	22	30	36
Drive Alone Mode Share (%)	80	79	78	76	73
Journey to Work time ( 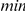 )	23.4	24.4	25.0	27.0	29.9
VKT per capita ( 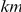 )	41.12	42.24	38.63	38.80	37.22
Accessibility (*Jobs in 30 minutes*)	1120460	1029221	1136220	1291576	1491337
Urbanized Area Density ( 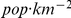 )	776	764	951	983	1103
MSA Population	1200929	1666799	2174582	3511888	8014309
Median HH Income	42733	46441	43755	49309	48811

As can be seen, connectivity measured in a variety of ways (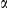, 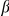, 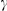, 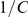, 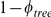) increases with metropolitan area population. If we imagine a city growing radially out from a point, as it gets larger, it connects the radial elements with cross-routes. This happens fractally, for major facilities as beltways are built, and for smaller roads as infill development occurs. Thus larger areas are less dendritic and more web-like. Larger metropolitan areas are also more likely to be polycentric. The entropy declines with city size as there is somewhat less variety in road types, meaning there are a greater share of low-level roads, and fewer high-level roads (freeways). Larger areas also (and not surprisingly) have fewer overall meters of roadway per capita, as the population density can increase faster than network density, as it is relatively easy for residential structures to increase vertically, while it is much more expensive for transportation facilities to do the same. Large cities also suffer more delay.

Perhaps surprisingly average edge length (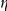) increases with city size. This may be due to larger metropolitan areas having a greater spatial extent (including relatively more suburban and exurban areas) with longer road segments. Not surprising, traffic per vertex (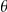) increases. Circuity seems largely invariant to city size. Large cities are less dendritic. As the city becomes larger, it becomes more connected, but has longer journey to work times, while larger cities have less capacity per capita.

**Figure 2:**
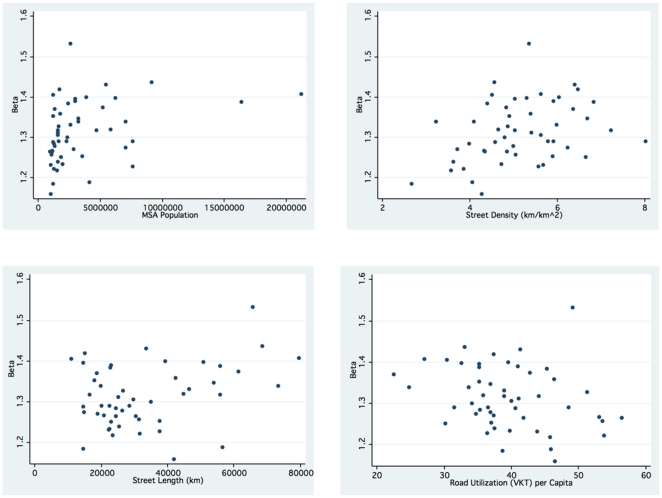
Beta vs. Population (slope = 0.0297, 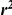
 **= 0.11,** 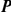 **= 0.018), Street Length (slope = 0.0421,** 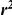 **= 0.10,** 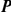 **= 0.023), Street Density (slope = 0.0991,** 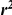 **= 0.11,** 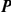 **= 0.006), Road Utilization per Capita (slope = −0.0825,** 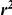 **= 0.07,** 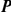 **= 0.06).** Network connectivity (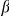) increases with population, road length, and road density, but declines with personal travel.

For US networks, the maximum observed Beta is just above 1.5, indicating the typical intersection is 3-way. 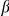 grows with population, network size, network density, but is correlated with lower road utilization per capita (Figure 2). This suggests that it is not only population density which results in less driving, but also more connected street (and by implication pedestrian) networks. The sources of lowered driving include more direct trips and increased non-auto use.

### Scaling rules

Following [14], [49] and others, a power law is used to describe how network structure and performance variables depend on city size.
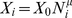
(12)


This can be transformed with natural logs to estimate with a linear regression:
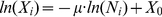
(13)where 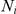 is the independent variable, which in this analysis we take to be metropolitan area population (city size), and 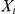 is the dependent variable, which are a variety of network structure variables (coefficient 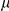 and normalization constant 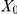 are to be estimated).

The estimated coefficient (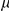) indicates whether sublinear or superlinear scaling obtains. The models are estimated using OLS regression.

For the models estimated in Table 2, all are sublinear, indicating a 1 percent increase in metropolitan population leads to less than a 1 percent change in the dependent variable. All of the variables are statistically significant at the 95 or 99 percent confidence level except for circuity, which is statistically insignificant. For instance, the individual regressions indicate that for every 1 percent increase in population, 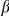 increases by 0.03 percent. The net result is that network connectivity increases with city size. The most significant measured variable is 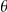, which is an indicator of traffic.

**Table 2:** Regressions: Independent Variable = ln(MSA Population).

	ln(MSA Pop.)		Constant		Adjusted
Dependent Variable (ln)	Coefficient	t-stat	Coefficient	t-stat	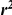
alpha ( 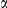 )	0.126	2.98	−3.75	−5.96	0.14
beta ( 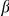 )	0.0296	3.01	−0.164	−1.13	0.14
gamma ( 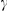 )	0.0296	3.01	−1.26	−8.69	0.14
eta ( 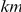 ) ( 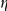 )	0.246	4.04	−6.11	−6.80	0.24
theta ( 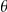 )	0.392	6.22	−6.11	−6.57	0.44
Circuity ( 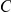 )	−0.00876	−0.85	0.39	2.63	0.00
Treeness ( 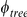 )	−0.151	−2.87	0.791	1.02	0.13
Entropy ( 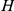 )	−0.190	−3.49	1.98	2.47	0.19
Roadways ( 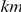 )	0.667	9.51	−0.439	−0.42	0.65
Roadways per capita ( 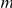 )	−0.219	−4.6	5.00	7.22	0.30
Travel Time Index	0.080	7.26	−0.96	−5.92	0.51
Drive Alone Mode Share	−0.713	−6.50	5.46	33.72	0.47
Journey to Work time ( 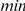 )	0.109	8.20	1.63	.833	0.57
VKT per capita ( 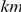 )	−0.129	−3.48	−1.22	−2.24	0.19
Accessibility ( 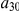 )	0.213	4.20	10.81	14.41	0.26
Urb. Area Density ( 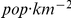 )	0.283	4.97	2.57	3.06	0.30
Median HH Income	0.094	4.66	9.35	31.57	0.30

The results indicate larger cities have different network structures than smaller cities. Their networks have more connections and are less dendritic. From the perspective of enabling intra-urban interactions, they are more efficient structures than those found in smaller cities. While this result can only establish correlation not causation, it is suggests two alternative (and not mutually exclusive) hypotheses that either well-connected networks help city growth, and that large cities construct more connected networks as the value of additional links is higher. Clearly future research is required to establish whether (or under what conditions) one or both of these explanations hold.

We can also use this data to test previous findings. [50] write “Quantities reflecting wealth creation and innovation have 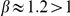 (increasing returns), whereas those accounting for infrastructure display 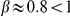 (economies of scale).” This research finds that median household income does increase with city size. A one percent increase in size leads to a 0.09 percent increase in household income, which is in the same direction, but a somewhat different magnitude than previous research.

Similarly these results corroborate the findings of [50] and others about sublinear infrastructure scaling. Each 1 percent increase in population leads to only a 0.67 percent increase in roadways. Further, each 1 percent increase in population reduces roadways per capita by 0.21 percent (Table 2).

It is not argued that any of these variables depend only on city size (in fact the regressions demonstrate they most certainly don't), but rather to find our what this relationship is as a means of exploring the extent to which city size is a factor. Clearly other factors could be at play. For instance, age of road network elements and geographic location may present important factors explaining network structure, in particular different design standards are in force at different times of history (which occurs in all cities, but obviously earlier design standards affect older cities more while newer design standards disproportionately affect younger cities. Similarly topography matters, whether a city is hillier or whether a city is on a large body of water affect how network structure is realized. Further population density, which depends in part on city size, may affect network structure.

### Rankings

The rankings of accessibility across US cities are shown in Table 3, which lists metropolitan areas by their largest or primary city.

**Table 3:** American Metropolitan Areas Ranked by Accessibility to Jobs by Automobile in 2010.

Rank	10 min	20 min	30 min	40 min	50 min	60 min	Weighted Average
1	Salt Lake	San Jose	San Francisco	Los Angeles	Los Angeles	New York	Los Angeles
2	Columbus	San Francisco	Los Angeles	New York	New York	Los Angeles	New York
3	San Jose	Columbus	New York	Washington	Chicago	Chicago	San Francisco
4	Grand Rapids	Las Vegas	Phoenix	Miami	Dallas	Boston	Washington
5	Raleigh	Los Angeles	Washington	San Francisco	Philadelphia	Dallas	San Jose
6	San Francisco	Salt Lake	Miami	Houston	Boston	Philadelphia	Phoenix
7	Las Vegas	Raleigh	Minneapolis	Philadelphia	Washington	Washington	Miami
8	Los Angeles	Milwaukee	Denver	Dallas	Houston	Houston	Salt Lake
9	Milwaukee	Riverside	Baltimore	Chicago	Atlanta	Atlanta	Chicago
10	Riverside	New York	Riverside	Minneapolis	Miami	Miami	Columbus
11	New York	Austin	Pittsburgh	Phoenix	San Francisco	San Francisco	Dallas
12	Honolulu	Phoenix	Houston	Boston	Detroit	Detroit	Philadelphia
13	Austin	Charlotte	Philadelphia	Detroit	Minneapolis	Minneapolis	Houston
14	Phoenix	Cleveland	Dallas	Atlanta	Phoenix	Phoenix	Minneapolis
15	Charlotte	Orlando	Chicago	Seattle	Seattle	Seattle	Las Vegas
16	Cleveland	Washington	Cleveland	St. Louis	St. Louis	St. Louis	Riverside
17	Oklahoma City	Grand Rapids	Portland	San Diego	San Diego	San Diego	Boston
18	Orlando	San Antonio	Cincinnati	Denver	Denver	Denver	Raleigh
19	Washington	Miami	Orlando	Baltimore	Baltimore	Baltimore	Denver
20	Rochester	Nashville	San Jose	Riverside	Riverside	Riverside	Baltimore
21	San Antonio	Portland	Kansas City	Pittsburgh	Pittsburgh	Pittsburgh	Cleveland
22	Miami	Minneapolis	Boston	Tampa	Tampa	Tampa	Milwaukee
23	New Orleans	Denver	San Diego	Cleveland	Cleveland	Cleveland	Orlando
24	Nashville	Kansas City	St. Louis	Portland	Portland	Portland	Detroit
25	Portland	Baltimore	Detroit	Cincinnati	Cincinnati	Cincinnati	Pittsburgh
26	Minneapolis	Cincinnati	Tampa	Orlando	Orlando	Orlando	Charlotte
27	Denver	Oklahoma City	Las Vegas	San Jose	San Jose	San Jose	Portland
28	Kansas City	Memphis	Charlotte	Kansas City	Kansas City	Kansas City	Kansas City
29	Baltimore	Pittsburgh	Milwaukee	Las Vegas	Las Vegas	Las Vegas	Cincinnati
30	Cincinnati	New Orleans	Columbus	Charlotte	Charlotte	Charlotte	Austin
31	Memphis	Houston	Indianapolis	Indianapolis	Indianapolis	Indianapolis	St. Louis
32	Pittsburgh	Rochester	San Antonio	Milwaukee	Milwaukee	Milwaukee	Grand Rapids
33	Houston	Buffalo	Atlanta	Columbus	Columbus	Columbus	San Diego
34	Buffalo	Philadelphia	Nashville	San Antonio	San Antonio	San Antonio	Atlanta
35	Philadelphia	Dallas	Seattle	Nashville	Nashville	Nashville	San Antonio
36	Dallas	Louisville	Salt Lake	Salt Lake	Salt Lake	Salt Lake	Nashville
37	Louisville	Chicago	Sacramento	Sacramento	Sacramento	Sacramento	Tampa
38	Chicago	Jacksonville	Raleigh	Raleigh	Raleigh	Raleigh	Seattle
39	Jacksonville	Providence	Austin	Austin	Austin	Austin	Oklahoma City
40	Providence	Hartford	Norfolk	Norfolk	Norfolk	Norfolk	Memphis
41	Hartford	Boston	Providence	Providence	Providence	Providence	Sacramento
42	Boston	San Diego	Hartford	Hartford	Hartford	Hartford	Indianapolis
43	San Diego	Sacramento	Louisville	Louisville	Louisville	Louisville	Providence
44	Sacramento	St. Louis	Memphis	Memphis	Memphis	Memphis	Rochester
45	St. Louis	Detroit	Jacksonville	Jacksonville	Jacksonville	Jacksonville	New Orleans
46	Detroit	Tampa	Grand Rapids	Grand Rapids	Grand Rapids	Grand Rapids	Louisville
47	Tampa	Norfolk	Oklahoma City	Oklahoma City	Oklahoma City	Oklahoma City	Hartford
48	Norfolk	Honolulu	Buffalo	Buffalo	Buffalo	Buffalo	Norfolk
49	Indianapolis	Indianapolis	New Orleans	New Orleans	New Orleans	New Orleans	Buffalo
50	Atlanta	Atlanta	Rochester	Rochester	Rochester	Rochester	Jacksonville

The final column shows that the five metro areas whose residents can reach the most jobs (weighted by travel time) are Los Angeles, New York, San Francisco, Washington, and San Jose. The rankings vary by the time threshold one is considering.

The tables may be surprising. Why are not some big cities (e.g. Chicago, Philadelphia, or Houston) better represented at the top? Keep in mind what is being represented here, the number of jobs reachable from an average point in the metro area by automobile, with more weight to jobs reachable in 10 minutes than 20 minutes, and more weight to jobs reachable in 20 minutes than 30 minutes and so on. Small cities show prominently in the 10 min accessibility threshold. These cities are both fast and compact, so their employment can be reached quickly. If I am interested in how many jobs I can reach in 10 minutes of driving, I am better off in Salt Lake than New York, since I can get to many of Salt Lake's jobs readily, but few of New York's.

When we use a 60 minute threshold, this list looks very much like the list of employment by metro area (as in 60 minutes, almost everyone can reach (nearly) every job in every metro). But within 30 minutes, the density of jobs and the speed of the network are both quite important. While the number and density of jobs is tending to increase as cities become more populous (and most of the top 50 cities are growing in this period), the speed on the network is declining as traffic growth outpaces network investment. Whether job density is growing faster than speed is declining depends on the case, and as can be seen by comparing various cities by year, there is a wide dispersion.

### Journey to Work

In 2011 there were 131 million people employed in the United States, most of whom commute to work regularly [51]. The Journey to Work (and the Return Home) remains a defining trip organizing spatial structure in metropolitan areas. Homes and workplaces are located relative to each other in order to keep the journey to work at acceptable levels while enabling workers to choose to desirable homes. Time spent traveling is a critical factor for what we might think of as personal productivity, and clearly has value, as land is far cheaper at the edges of metropolitan areas than toward its center. This suggests most Americans would prefer their commute were shorter, faster, and less congested, all else equal. The average Journey to Work travel time varies considerably across US cities. What are the causes of such variance?

It has long been observed that more populous cities have longer commutes. There are several possible reasons for this. More populous cities tend to have more congestion because it is easier to add people per unit area than transportation services. We have data on congestion levels, and so can control for this.

Larger cities also have more opportunities, so travelers can travel longer and still remain within the region. While in a small city, a 30 minute trip might leave the region of available jobs, in a large city, one might still be in the area. So given some possibility of taking a job at a long distance vs none, the larger city, with some possibility at a longer distance should pull the average rightward (higher).

Another hypothesis that has been broached is economies of agglomeration. In a large city there is greater specialization and division of labor for a variety of reasons. This specialization may lead to people having only a few possible work location to work in a specialized jobs (at the extreme, e.g. only three Professors of Transportation Engineering are located in metropolitan Minneapolis/St. Paul, all in adjacent offices), if people located randomly, specialization would lead to longer commutes, or if spouses were equally but differently specialized, that might also lead to longer commutes. We have no reason to believe people would be randomly located though, so the effects of this are ambiguous.

Large cities tend to be denser on average, but in contrast with the increase in area a large city brings (which would tend to expand the average journey to work), the density should on average shorten the journey to work, as things are closer together. Accessibility, as defined above, directly measures how many destinations can be reached per unit time. This differs from conventional density measures, which measure how many destinations are available per unit area.

Choice of mode might matter. Some modes are more efficient than others at getting from place to place in a short time. In particular, in most US cities, highways are faster for most point-to-point trips, while transit, with schedule delays, circuitous routes (compared with the true origin and destination), and many stops (especially for local services) is usually longer. The more people who take transit, the higher the journey to work time.

The connectivity of the network might also play a role. Connectivity has not been previously analyzed with regards to the journey to work. The more connected the network is, the more direct it is. The alpha index (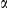) is the ratio of the actual number of circuits in a network to the maximum possible number of circuits in that network. Values range from 0 percent (no circuits) to 100 percent (a completely interconnected network). Real networks are neither perfect, nor planar, nor grids, though they may approximate them.

Finally income may affect willingness to travel, as I am both more willing to travel for a higher job, I may face more congestion if other people are wealthier, and I may have different preferences for travel relative to other amenities. The literature has traditionally found the amount of travel increases with income.

The results of Table 4 are entirely consistent with the theory laid out above. Since this is essentially a log-log model, the elasticities can be read directly from the coefficients. For instance a 1 percent increase in population will lead to a 0.114 percent increase in journey to work time.

**Table 4:** Dependent Variable 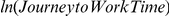.

	Coef.	Std. Err.	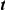	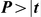
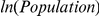	0.100	0.0165	6.06	0.000
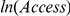 ( 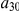 )	−0.0589	0.0300	−1.96	0.056
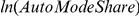	−0.405	.133	−3.03	0.004
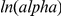 ( 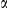 )	−0.0999	0.0356	−2.78	0.008
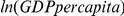	0.0536	0.0326	1.64	0.108
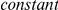	2.322	0.436	5.32	0.000
Adjusted 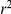	0.7417			
N	49			

The table also means that a 1 percent increase in accessibility reduces average metropolitan commute times by 0.0612 percent or about 90 seconds each way. That might seem small, but for a typical 25 minute commute, twice a day, 250 work days a year, over 40 years, that amounts to 510 hours (or 21 full days) per person. For a city with a million people, this is 58,219 person years over 40 years (or 1,455 person years per year).

Accessibility changes by well more than 1 percent (up or down) per decade in most metropolitan areas, so this kind of change is quite feasible.

In brief:

- More populous cities have longer commutes (due to the greater opportunities available).
- More accessible cities have shorter commutes (due to the more spatially efficient arrangement of activities).
- Transit cities have longer commutes (due to the longer access and waiting costs of transit networks).
- Connected cities have shorter commutes (due to the more efficient network for travel).
- Wealthier cities have longer commutes (due to different preferences for amenities and more non-work travel congesting the roads).

### Automobile Mode Share

Accessibility and network structure is not important just for those who use a car. The same factors that affect accessibility by auto affect accessibility by other modes (transit, walking). Most US transit use is by bus, and bus speeds depend in part on highway speeds. Thus accessibility can be used to help determine what share of the population will use a car on their Journey to Work. We can predict metropolitan mode share by auto (drive alone plus carpool) as function of accessibility, city size, income, and network structure.

An increase in auto accessibility without a concomitant increase in non-auto accessibility might be thought to increase auto share (the car would be more attractive). However these factors do not move independently. The same cities with a high auto accessibility also have a high transit accessibility. The high density that is a factor in both makes transit more effective. Other hypotheses are as follows:

- Population increases congestion, and makes the auto a less viable mode than alternatives.
- Wealthier cities can afford to invest more in transit and other modes, and make them more viable alternatives.
- How treelike or dendritic (as opposed to mesh-like) the network is, clearly discourages auto travel, as it makes travel more difficult. But a treelike structure aids transit, especially on trips toward the center, as transit often functions in a treelike manner itself.

The model (Table 5) implies a 1 percent increase in Access will reduce metropolitan average driving mode share by 0.0575 percent. This again may seem small, but if driving mode share is 90 percent for a typical metro area, a 1 percent increase in accessibility resulting in a 0.0575 percent drop in auto mode share reduces it to 89.48 percent. This implies that non-auto mode share increased from 10 percent to 10.52 percent (a 5.2 percent increase).

**Table 5:** Dependent Variable 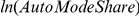.

	Coef.	Std. Err.	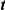	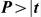
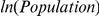	−0.0905	0.0128	−7.03	0.000
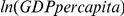	−0.0646	0.0345	−1.87	0.068
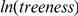 ( 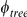 )	−0.0578	0.0316	−1.83	0.075
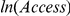 ( 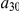 )	−0.0609	.0317	−1.92	0.061
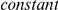	1.505	0.398	3.78	0.000
Adjusted 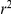	0.5048			
N	49			

## Discussion

Accessibility to jobs by car is not the only thing people care about. If it were, cities would be packed together on a minimum amount of space so people could live on top of their job, or everyone would work at home. Measuring (and then valuing) access to other characteristics, and considering the trade-off between that and space for living is a central problem of urban economics, regional science, and planning. While being more accessibility is generally better, all else equal; all else is seldom equal. There are costs as well as benefits associated with accessibility. If the price of land is higher, I can afford less of it. If I travel by car, streets in places with more activities are inherently more crowded, and my car trips are less pleasurable. If I travel by transit, I have less privacy than by car, and so on.

This research provides a new methodology and dataset to enable inter-metropolitan comparisons of accessibility in a way that is clearly understood and explainable, that tracks with our experience and the available evidence, and that does not require complex mathematical calculations.

An improved understanding of urban structure requires progress beyond simple land use variables to consider the underlying network pattern. This paper explores a set of road network structure variables and examines how they affect a variety of other network performance measures, and how they are affected by population. This research corroborates previous findings that larger cities have more delay, longer commutes, and less travel per person. It also finds that larger cities have more connected road networks, corroborating similar findings about transit networks [4], are more accessible, and are less hierarchical (in terms of network hierarchy).

We may be able to explain earlier findings that larger cities have more wealth and innovation per capita hold up because of the efficiency of intra-urban connectivity that larger cities bring. It might not only be the potential for contacts, but also the efficiency of interaction (and hence the number of contacts per unit time, and the amount of time spent with contacts rather than in transport) that brings about that super-linear scaling.

Alternatively, the causality may be reversed, as cities grow the agents within them naturally create more connected networks to maximize local gains, but those network elements may dampen collective wealth creation instead of reinforcing it. More connected and less tree-like networks may have less focus on a single downtown, and consequently may lower the economies of agglomeration that depend on face-to-face interaction and serendipitous interactions. The resolution to this depends very much on the scale on which economies of agglomeration operate, and may vary by industry.

The question remains, and should be the subject of future study, whether the internal spatial structure of cities causally determines their ultimate productivity. Teasing-out this relationship may be difficult, but is important as to whether (or to what extent) the gains of efficient urban networks are captured by residents in terms of time savings and higher quality of life (e.g. more space), or translate into more conventionally measured economic output. These 50 cities act as a laboratory, each engaging in different network investment and land use strategies, and resulting in different accessibilities that affect their transportation and economic performance. Additional future work should examine other cities, including smaller cities (below the top 50) and cities in other countries, to test whether the relationships identified in this paper hold.

This research has several implications for urban planning and management. While networks are persistent features of cities (an urban traveler from hundreds of years ago may be able to comfortably navigate the old part of his home town today), the network structure of cities as a whole likely is not, as newer areas may have different topological structures than older areas, just as larger cities differ from smaller cities and the core differs from the periphery. In the absence of conscious intervention, network structures will evolve over time serving the needs of the relevant decision-making agents. Planners however can intervene to make cities more (or less) inter-connected through design rules [52] and investment decisions [53]. This will have implications for resultant urban accessibility, how individuals use cities, the scope of their activity space [54], their resultant travel behavior, and ultimately the economic activity in the city, as potential agglomeration economies are exploited or allowed to whither.

There are many improvements to be made, including calculating accessibility within each city using detailed network and land use data, rather than metropolitan averages. This requires considerably more data (an accurate estimate of travel times between each origin-destination pair) and computation, but should produce a more accurate result. Computing accessibility for other modes (e.g. [55]), and to other purposes are also natural extensions.
